# The relationship between inflammatory biomarkers and macular pigment optical density in hypertensive patients: a cross-sectional study

**DOI:** 10.2478/abm-2023-0054

**Published:** 2023-10-09

**Authors:** Chunchuree Kongmeesuk Kaneko, Katsunori Kaneko, Vitoon Jularattanaporn, Thamthiwat Nararatwanchai

**Affiliations:** 1 School of Anti-Aging and Regenerative Medicine of Mae Fah Laung University, Bangkok 10110, Thailand; 2 Graduate School of Economics, Osaka Metropolitan University, Osaka 558-8585, Japan

**Keywords:** hsCRP, hypertension, inflammatory biomarker, macular pigment optical density, macular pigment screener II

## Abstract

**Background:**

Inflammation may be associated with macular pigment optical density (MPOD) degradation.

**Objectives:**

The relationship between inflammation and MPOD is evaluated using inflammatory biomarkers, including high sensitivity C-reactive protein (hsCRP), lipid level and ratio, waist circumference (WC), and body mass index (BMI).

**Method:**

In this cross-sectional design, 62 hypertensive patients were recruited between January 6 and January 8, 2022, at a primary care unit. The MPOD was measured using the Macular pigment screener II. Blood tests for hsCRP, lipid profile, WC measurement, BMI calculation, and completing a questionnaire were conducted, and statistical analysis was done by using Microsoft Excel 2019 and Stata version 16.1. Spearman's rank correlation test was used to evaluate correlations. Multivariate analysis for adjusting confounders was done by logistic regression.

**Result:**

There was a significant negative correlation between hsCRP >3 and MPOD (*r* = −0.26, *P* = 0.04).

**Conclusion:**

Inflammation was linked to MPOD. Anti-inflammatory agents may be beneficial in preventing MPOD degradation.

Age-related macular degeneration (AMD) is a leading cause of blindness among the elderly worldwide. AMD has a wet and dry form, and the dry form's cause is not yet fully understood. Yet, an online assessment of the PubMed database from 1980 to date revealed that smoking, nutritional factor, lipids, cardiovascular disease, age >60 years, and family history are major risk factors affecting AMD. There are presently no effective treatments for dry AMD. As a result, the focus is on risk factors for disease prevention [1]. A study revealed a significant association between decreased macular pigment optical density (MPOD) levels and drusen maculopathy among participants aged 45–65 years. MPOD levels were 0.41, 0.31, and 0.17 in 160 patients with AMD stages 1, 2, and 3. As a result, decreased MPOD becomes a risk factor for AMD. A macular pigment composes of 2 carotenoids, lutein and zeaxanthin, with absorption spectra in the 400–540 nm spectral range and maximal absorption of 460 nm. Because of its ability to absorb blue light and scavenge free radicals, the macular pigment may help prevent AMD [2]. MPOD reflects the amount of lutein and zeaxanthin in the macular pigment region related to the ability of the macular pigment to absorb blue light. Optical density levels, or density units (d.u.), range from 0 to 1 and can be measured by Macular pigment screener II (MPS II). MPS II is the first and most frequently used noninvasive method for quantifying central macular pigment levels and is a psychophysical method such as heterochromatic flicker photometry (HFP) by presenting 2 light stimuli of varying wavelengths to the individual in the form of a flicker. The light stimuli alternate between blue light that is absorbed by the macular pigment and green light that is not absorbed by the macular pigment in the retina. It begins with flashes of 2 different lights directed at the fovea (standard mode). Gradually, the light will dim, and the patient will reply by reporting the smallest flashing light. Then, MPOD was calculated by computer using the log ratio and reported as accept, caution, or reject. The Macular Pigment Consensus Panel classified central MPOD levels into 3 categories: low, mid-range, and high. MPOD levels <0.2 d.u. (low), 0.2–0.5 d.u. (mid-range), and >0.5 d.u, (high). In order to prevent MPOD degradation, MPOD levels below 0.5 d.u. should be supplemented [3]. Additionally, a study indicated that the MPS II technique could be used in future investigations to examine MPOD accurately [4]. It has been found that there are higher MPOD levels in healthy people than in AMD patients. Increased MPOD is associated with an enhanced visual performance. Lutein or zeaxanthin supplementation aid in increasing MPOD and can reduce the risk of AMD. However, MPOD is affected by several other factors related to risk factors for AMD [3]. To prevent the degradation of MPOD, researchers are interested in whether inflammation may affect MPOD degradation. Thus, this study aims to examine the relationship between inflammation and MPOD using inflammatory biomarkers as follows in hypertensive patients who have been linked to an inflammatory condition [5].

High sensitivity C-reactive protein (hsCRP) is a sensitive blood test that detects a lower level of C-reactive protein (CRP) compared to the conventional CRP measurement and is quantified in mg/L. However, there are currently no highly effective markers to diagnose chronic inflammation. In addition to the diagnosis, when inflammation is associated with clinical symptoms, blood tests of systemic inflammation include hsCRP, fibrinogen, and pro-inflammatory cytokines like tumor necrosis factor-alpha, interleukin-1 beta, interleukin-6, and interleukin-8 [6]. In daily practice, the inflammatory biomarker in the broadest used to help predict the risk of developing CVD is hsCRP. Data also support the role of inflammation in cardiovascular disease and other diseases characterized by inflammation [7]. Thus, this study used hsCRP as the inflammatory biomarker. In addition, inflammation has been identified in individuals who have insulin resistance or are obese. As detailed below, numerous data, such as lipid levels and ratios, waist circumference (WC), and body mass index (BMI), have been used to indicate inflammation.

WC is the measurement taken at the middle of the iliac crest and the bottom of the ribcage according to WHO guidelines, which are appropriate for the Asian population, are for WC ≥90 cm (men) or ≥80 cm (women) to classify abdominal obesity, which increases the CV risk and metabolic syndrome [8]. They also imply that central obesity is a key marker of the inflammatory state and contributes to an increased risk of central obesity and metabolic syndrome development [9].

BMI is calculated by weight divided by height in kg/m^2^. It is used to classify underweight, overweight, and obesity. Weight status varies by ethnicity. The study discovered a more appropriate relationship between obesity and illness manifestations in the Asia-Pacific Classification than in the WHO Classification, even though the BMI cut-off of the Asian-Pacific Classification is lower by normal range (18.5–22.9), overweight (23–24.9), and obese (≥25) [10]. BMI is a significant marker to predict inflammation in the general population. The investigation discovered that BMI might affect the inflammatory profile of critically ill sepsis patients [11].

Lipid profile is a measurement of the amount of fat in the blood that includes total cholesterol (TC), triglycerides (TG), high-density-lipoprotein cholesterol (HDL-C), and low-density lipoprotein cholesterol (LDL-C) in mg/dL. The lipid ratio is a proportional measurement of fat in the blood that can predict illness risk. Several studies have found an association between lipid level and ratio with inflammation in the body. The lipid ratio of TC/HDL-C is occasionally used instead of total blood cholesterol, where the ideal level is 3.5:1. As for middle-aged women, TC/HDL-C≤3.5 has the lowest hazard ratio for acute myocardial infarction and can be used as a clinical predictor in early screening of AMI [12]. When a standardized insulin assay is lacking, 3 relatively basic metabolic markers can help identify insulin resistance. For obese individuals at risk of insulin resistance, most clinics recommend a TG concentration of 1.47 mmol/L (130 mg/dL) or TG/HDL-C (>3), and insulin concentrations (109 pmol/L) to help identify insulin resistance and increase the risk for various adverse outcomes [13]. From the above studies, inflammatory biomarkers, including hsCRP, lipid level or ratio, BMI and WC, are related to inflammation and applied in clinical practice. Therefore, we used them to find the association of inflammation with MPOD in this study.

## Method

The cross-sectional study was conducted in a Ban Kongdara Primary Care Unit in Sriracha District, Chonburi Province, Thailand, from January 6 to 8, 2022. The study was approved by Mae Fah Luang University Ethics Committee on Human Research (Certificate of approval No. EC 21177-20). Total correlation sample size was calculated a priori as n = [(Z_α_ + Z_β_)/C]^2^ + 3 = 62 [14] in order to achieve α = 0.025 (2-tailed) (Error rate of type I), b = 0.20 (Rate of Type II errors). The predicted correlation coefficient is *r* = 0.38 from our pilot study, which is the same as this study at Ban Kongdara Primary Care Unit because there has never had research on this before. Z_α_ = 2.2414, Z_β_ = 0.8416, C = 0.5 * ln[(1+r)/(1−r)] = 0.4001.

The participants were recruited by inclusion criterion (1) hypertension alone or in conjunction with other diseases; (2) aged 35–59 years. Because of AMD and the reduction of MPOD, it is more common in people over 60 years of age. Therefore, the research was conducted in the age group younger than 60 years to find factors to prevent or slow the disease. In addition to this primary care unit, the hypertensive clinic had the youngest patients at age 35 years. Exclusion criterion were (1) participants who consume lutein and zeaxanthin, (2) individuals with mild to severe medial opacity (corneal scar, cataract), and who have infection or inflammation, and (3) individuals who have AMD or other ocular illnesses such as glaucoma, optic atrophy, cataract, previous retinal laser treatment or surgery, or previous eye damage. Withdrawal criterion were (1) participants with problems in using the MPS II device, (2) 3 consecutive failures of the MPS test, and (3) participants who had not completed both the blood test and the MPOD measurement. Physical examinations are used as exclusion criteria. Prior to the start of the study, all participants received a research consent form and an information leaflet to sign in, and patients were requested to complete questionnaires on MPOD. All participants received an explanation of how to conduct the MPS test before starting the test. Then, participants had blood checked for hsCRP, had a lipid profile after fasting for 9–12 h, and were measured for blood pressure, WCs, and calculated BMI. For each person, it takes about 15 min to test both eyes and explain the results of the MPS test. In the case of infection with COVID-19, participants were divided into 3 groups to measure MPOD, and it took 3 days. The 69 OPD cardholders met the inclusion criteria and were invited to participate in the study. But 3 patients missed their appointments due to illness, 2 had corneal scarring revealed during physical exams, and 2 failed the MPS II test 3 times. Finally, there remained 62 participants in the study. Computerized statistical analysis was done using Microsoft Excel 2019 and Stata version 16.1(license serial number:401606287634). Descriptive data analysis of the basic parameters were reported in mean ± SD., Min. and Max. Spearman's rank correlation (*rs*) was used to examine the relationships between biomarkers and MPOD. Multivariate analysis for adjusting confounders was done by logistic regression.

## Result

Of 62 participants, 27 (43.55%) were male and 35 (56.45%) female. The sex ratio was 1: 1.30 There were obese people with a BMI over 25 (70.96 %) and smoking 17 (27.42%). The basic characteristics of participants by mean ± SD, Min & Max. are shown in **Table 1.** In this study, the mean estimated MPOD of hypertension was 0.48 ± 0.14 in the mid-range. **Table 2,** there was a significant negative correlation (*P* < 0.05) between hsCRP > 3 and MPOD (*r* = −0.26, *P* = 0.04) by Spearman's rank correlation. Linear regression was performed, but there was no linear relationship. BMI, waist circumference, lipid profile, and ratio had no significant correlation. Multivariate analysis of the associated factors between MPOD by logistic regression in **Table 3**. There were no significant. The associations between sex, smoking, and MPOD were not significant when using MPOD < 0.5 [3] by chi-square analysis in **Table 4.**

**Table 1.** Basic characteristics of participants by mean ± SD, min. and max.

**Parameters (n = 62)**	**Mean ± SD**	**Min.**	**Max.**
Systolic blood pressure (mm/Hg)	138.77 ± 13.97	104	169
Diastolic blood pressure (mm/Hg)	96.68 ± 14.09	65	129
Duration of hypertension(years)	4.07 ± 4.94	0.16	26
Body Mass Index (kg/m^2^)	27.49 ± 5.46	17	42.9
Waist Circumference (cm)	92.00 ± 14.23	64	150
hsCRP (mg/L)	2.70 ± 3.35	0.2	18.6
TC (mg/dL)	192.31 ± 40.91	108	283
HDL-C(mg/dL)	52.21 ± 15.87	30	114
LDL-C (mg/dL)	118.79 ± 37.60	52	213
Triglyceride (mg/dL)	161.26 ± 133.92	43	1055
TC/HDL-C	3.88 ± 1.06	1.89	6.91
TG/HDL-C	3.50 ± 3.32	0.38	24.53
LDL-C/HDL-C	2.41 ± 0.87	0.84	4.94
Age (years)	52.66 ± 5.32	35	59
Right estimate MPOD (d.u.)	0.48 ± 0.15	0.19	0.9
Left estimate MPOD (d.u.)	0.49 ± 0.15	0.14	0.82
Mean estimated MPOD (d.u.)	0.48 ± 0.14	0.22	0.84

hsCRP, high sensitivity C–reactive protein; MPOD, Macular Pigment Optical Density; TC, total cholesterol; TG, triglyceride; HDL–C, high–density–lipoprotein cholesterol; LDL–C, low–density lipoprotein cholesterol

**Table 2.** The spearman's rank correlation coefficients(r_s_) mean MPOD between parameters.

**Parameters (n=62)**	**Mean MPOD (d.u.)**	

	***r***	***P***
hsCRP>3 (mg/L)	−0.26	0.04^*^
BMI (kg/m^2^)	−0.13	0.32
WC (cm)	−0.01	0.94
TC (mg/dL)	0.03	0.84
HDL–C (mg/dL)	0.17	0.20
LDL–C (mg/dL)	0.10	0.46
TG (mg/dL)	−0.19	0.13
TC/HDL–C	−0.10	0.44
TG/HDL–C	−0.18	0.17
LDL–C/HDL–C	0.01	0.98
Age (years)	−0.003	0.98
Duration of HT (years)	−0.06	0.66
SBP (mm/Hg)	−0.05	0.73
DBP (mm/Hg)	0.09	0.47

* *P* < 0.05.

hsCRP, High sensitivity C-reactive protein; BMI, Body Mass Index; WC, waist circumference; TC, total cholesterol; TG, triglyceride; HDL–C, high–density–lipoprotein cholesterol; LDL–C, low–density lipoprotein cholesterol; HT, hypertension; SBP, systolic blood pressure; DBP, diastolic blood pressure; MPOD, macular pigment optical density.

**Table 3.** Multivariate analysis with mean MPOD by logistic regression.

**Model 1** **MPOD (d.u.)**	**OR**	**Std. Err**	**z**	**P>|z|**	**95% CI**	**Model 2** **MPOD (d.u.)**	**OR**	**Std. Err**	**z**	**P>|z|**	**95% CI**
hsCRP >3(mg/L)	0.34	0.25	−1.45	0.15	0.08–1.45	hsCRP>3(mg/L)	0.28	0.25	−1.67	0.10	0.07–1.25
BMI (kg/m^2^)	1.07	0,13	0.56	0.57	0.85–1.35	BMI (kg/m^2^)	1.09	0.13	0.67	0.50	0.85–1.38
WC (cm)	0.98	0.05	0.05	0.69	0.90–1.08	WC (cm)	0.98	0.05	−0.54	0.59	0.89–1.07
TC (mg/dL)	0.98	0.02	−0.53	0.60	0.94–1.03	TC/HDL–C	0.41	0.62	−0.59	0.56	0.02–7.88
HDL–C(mg/dL)	1.02	0.03	0.82	0.97	0.97–1.09	TG/HDL–C	0.89	0.20	−0.53	0.60	0.57–1.38
LDL–C(mg/dL)	1.01	0.02	0.52	0.97	0.97–1.06	LDL–C/HDL–C	1.95	3.06	0.42	0.67	0.09–42.44
TG (mg/dL)	0.99	0.01	−0.70	0.99	0.99–1.01						
Age (years)	1.04	0.06	0.64	0.53	0.93–1.16	Age (years)	1.03	0.06	0.56	0.58	0.92–1.15
Smoking	0.98	0.66	−0.33	0.98	0.26–3.68	Smoking	1.07	0.73	0.11	0.91	0.29–4.05
Sex (Female)	0.32	0.24	−1.52	0.13	0.07–1.40	Sex (Female)	0.27	0.21	−1.68	0.09	0.06–1.24
SBP (mm/Hg)	1.01	0.02	0.84	0.40	0.98–1.06	SBP (mm/Hg)	1.02	0.02	0.87	0.38	0.98–1.06
Duration of HT (years)	0.97	0.07	−0.38	0.70	0.86–1.11	Duration of HT (years)	0.98	0.07	−0.36	0.71	0.86–1.11
_cons	0.03	0.13	−0.77	0.44	2.73–262.33	_cons	0.38	1.77	−0.21	0.84	0.0004–3322.67

* *P* < 0.05

OR, Odds ratio; Std. Err., standard error; CI, confidence interval; hsCRP, high sensitivity C-reactive protein; BMI, Body Mass Index; WC, waist circumference; TC, total cholesterol; TG, triglyceride; HDL–C, high–density–lipoprotein cholesterol; LDL–C, low–density lipoprotein cholesterol; HT, hypertension; SBP, systolic blood pressure

**Table 4.** The association between sex, smoking and mean MPOD.

**Parameters (n = 62)**	**Mean MPOD (d.u.)**			

	**<0.5 (d.u.)**	**≥0.5 (d.u.)**	***X^2^***	***P***
Male	13	14	0.86	0.35
Female	21	14		
Smoking				
Yes	24	21	0.16	0.70
No	10	7		

## Discussion

Our estimated mean MPOD of hypertension was 0.48 ± 0.14, and it is below the mean MPOD of healthy people in Korea, which was mean ± SD = 0.59 ± 0.20 [15]. Previous studies reported that hypertension and inflammation are somehow connected; circulating inflammatory molecules are higher in hypertensive patients and their levels indicate the onset of hypertension. The research shows that chronic inflammation can be used as a predictor of hypertension incidence [5]. Therefore, inflammation may contribute to lower MPOD levels.

hsCRP is utilized as an inflammatory marker, and multiple prospective studies in healthy individuals have validated its ability to predict cardiovascular events. The American Heart Association and the Centers for Disease Control and Prevention defined risk groups based on hsCRP levels in 2015 as low risk (< 1.0 mg/L), average risk (1.0 to 3.0 mg/L), and high risk (> 3.0 mg/L) [16]. This study showed hsCRP > 3.0 mg/L(high risk) had a significant negative correlation between MPOD (*r* = −0.26, *P* = 0.04). hsCRP levels may be useful to screen or follow clinically to prevent MPOD degradation, which is a risk factor for AMD and should be studied in broader applications.

BMI and WC were used to classify obesity, which is related to persistent systemic inflammation. It is remarkable that body fat and serum inflammatory protein levels have a positive correlation. However, for the relationship of inflammation from obesity, both BMI and WC should be used together, particularly in abdominal obesity, because WC shows a greater correlation with central obesity inflammation [17]. The association between BMI and MPOD was found in the previous study [18].

Several studies have found an association between lipid level and ratio with inflammation in the body. The TC/HDL-C can be used as a clinical predictor in the early screening of AMI [12]. A study discovered a strong link between the high TG/HDL-C ratio and insulin resistance and suggested that it could be utilized as an indication of insulin resistance in the nonobese Chinese middle-aged and elderly population in clinical practice [19]. This study found that BMI, waist circumference, lipid profile and ratio had no significant correlation with MPOD.

This study did not have a control group because it was designed to only look at the association of inflammation with other variables without intervention. Futhermore, the initial design of this study looked at the relationship between inflammation and MPOD. Thus, it focuses on people who are more likely to get inflammation. Smokers (27.42%) were not left out of this, which is a limitation of this study that cannot separate the causes of inflammation in more detail. Although logistic regression was used to adjust the confounder, the result had no significance. Treatments such as anti-hypertensive drugs, lipid-lowering drugs, and the duration of treatment may have an effect on MPOD, which should be researched in detail and controlled for various variables in a case–control study in the future.

## Conclusion

Inflammatory biomarkers (hsCRP > 3.0 mg/L) in this study showed significant relationships with MPOD. This indicates that inflammation is linked to MPOD. hsCRP may be helpful to screen or follow clinically to prevent MPOD degradation, which is a risk factor for AMD, especially in patients with chronic inflammatory disease and should be studied in broader applications. Anti-inflammatory supplements such as omega3, an anti-inflammatory diet may play a role in preventing the reduction of MPOD in addition to the use of lutein and zeaxanthin to prevent the reduction of MPOD.

## References

[1] Al-Zamil WM, Yassin SA (2017). Recent developments in age-related macular degeneration: a review. Clin Interv Aging.

[2] Chandrasekera A, Jayarathne V, Fonseka D (2016). Relationship between macula pigment optical density and visual performances in patients with dry age related macular degeneration accompanied with macula drusens. Int J Ophthal.

[3] Bernstein PS, Delori FC, Richer S, van Kuijk FJ, Wenzel AJ (2010). The value of measurement of macular carotenoid pigment optical densities and distributions in age-related macular degeneration and other retinal disorders. Vision Res.

[4] Davey PG, Alvarez SD, Lee JY (2016). Macular pigment optical density: repeatability, intereye correlation, and effect of ocular dominance. Clin Ophthalmol.

[5] Pauletto P, Rattazzi M (2006). Inflammation and hypertension: the search for a link. Nephrol Dial Transplant.

[6] Ansar W, Ghosh S (2016). Inflammation and inflammatory diseases, markers, and mediators: role of CRP in some inflammatory diseases. Biology of C Reactive Protein in Health and Disease.

[7] Ridker PM, Silvertown JD (2008). Inflammation, C-reactive protein, and atherothrombosis. J Periodontol.

[8] Razi SM, Manish G, Keshav GK, Sukriti K, Gupta A (2016). Site or size of waist circumference, which one is more important in metabolic syndrome?. Int J Med Public Health.

[9] Ackermann D, Jones J, Barona J, Calle MC, Kim JE, LaPia B (2011). Waist circumference is positively correlated with markers of inflammation and negatively with adiponectin in women with metabolic syndrome. Nutr Res.

[10] Lim JU, Lee JH, Kim JS, Hwang YI, Kim TH, Lim SY (2017). Comparison of World Health Organization and Asia-Pacific body mass index classifications in COPD patients. Int J Chron Obstruct Pulmon Dis.

[11] Zampieri FG, Jacob V, Barbeiro HV, Pinheiro da Silva F, de Souza HP (2015). Influence of body mass index on inflammatory profile at admission in critically Ill septic patients. Int J Inflam.

[12] Calling S, Johansson S-E, Wolff M, Sundquist J, Sundquist K (2019). The ratio of total Cholesterol to high density lipoprotein cholesterol and myocardial infarction in Women's health in the Lund area (WHILA): a 17–year follow–up cohort study. BMC Cardiovasc Disord.

[13] McLaughlin T, Abbasi F, Cheal K, Chu J, Lamendola C, Reaven G (2003). Use of metabolic markers to identify overweight individuals who are insulin resistant. Ann Intern Med.

[14] Hulley SB, Cummings SR, Browner WS, Grady D, Newman TB (2013). Designing clinical research: an epidemiologic approach.

[15] Hong IH, Jung WH, Lee JH, Chang IB (2020). Macular Pigment Optical Density in the Korean population: a cross sectional study. J Korean Med Sci.

[16] Knight ML (2015). The application of high-sensitivity c-reactive protein in clinical practice: a 2015 update. U. S. Pharmacist.

[17] de Heredia FP, Gómez-Martínez S, Marcos A (2012). Obesity, inflammation and the immune system. Proc Nutr Soc.

[18] Lee HN, Ismail F, Sudarno R, Pei AL, Subrayan V (2020). Correlations among macular pigment optical density, central macular thickness, and body mass index. Int Eye Res.

[19] Yang Y, Wang B, Yuan H, Li X (2021). Triglycerides to high-density lipoprotein cholesterol ratio is the best surrogate marker for insulin resistance in nonobese middle-aged and elderly population: a cross-sectional study. Int J Endocrinol.

